# Dynamic service analytics capabilities and service firm performance: Exploring the mediating roles of adaptive and customer-linking capabilities

**DOI:** 10.1371/journal.pone.0338542

**Published:** 2026-02-06

**Authors:** Yanting Huang, Kai Zhang, Xuejiao Chen

**Affiliations:** 1 School of Business Administration, Zhongnan University of Economic and Law, Wuhan, Hubei, China; 2 School of Tourism and Physical Health, Hezhou University, Hezhou, GuangXi, China; 3 School of Information and Security Engineering, Zhongnan University of Economic and Law, Wuhan, Hubei, China; 4 School of Economics and Management, Hubei Minzu University, Enshi, Hubei, China; Shenzhen University, CHINA

## Abstract

Although service firms use dynamic service analytics capabilities (DSAC) to respond to dynamic customer demands, how these capabilities translate into performance remains unclear. Based on a sample of 338, this study explores the effects of DSAC on firm performance and the chain mediating roles of adaptive and customer-linking capability. The results reveal that DSAC enhance performance; adaptive capability partially mediate between DSAC and firm performance; adaptive capability partially mediate between DSAC and customer-linking capability; and customer-linking capability partially mediate between adaptive capability and firm performance. Adaptive and customer-linking capabilities form a continuous mediating path through which DSAC enhance service firm performance. The findings not only enrich the existing research on the relationship between DSAC and firm performance, but also provide an actionable path for service firms to enhance the effectiveness of DSAC, helping practitioners to be more flexible in responding to market changes.

## Introduction

With the iteration of digital technologies and the deepening of the experience economy, we are confronted with a market environment characterized by volatility, uncertainty, complexity, and ambiguity (VUCA). In such a context, all organizations are compelled to enhance their agility and responsiveness in order to be able to comfortably cope with change and construct competitive advantage [1]. It is in this context that dynamic capabilities theory (DCT) has emerged as a central theoretical framework for understanding how organizations respond to volatile situations [2] In the field of service research, the concept of dynamic service analytics capabilities (DSAC) [3,4], developed by Akter et al. has certainly advanced this topic and it is theoretically forward-looking. DSAC can be seen as a specific extension of dynamic capabilities in the service domain, as a higher-order capabilities that helps service organizations gain insights into their business environments, anticipate the evolution of trends, and make prudent decisions in near-real time.

Despite the potential of DSAC at the theoretical level, as well as the exponential growth of research around dynamic service analytics techniques (e.g., research on the use of artificial intelligence techniques to enable dynamic services) over the past decade [5–7], a fact that should not be overlooked is the fact that research on DSAC at the strategic level has come to a standstill in recent years. This disconnect is alarming because when the rapid evolution of technology is not synchronized with the iteration of strategic frameworks, it often leads to a lack of guidance in practice, making it difficult for managers to effectively invest in DSAC. Further, there are still two key gaps in the current DSAC research: first, the cross-cultural structural validity of the higher-order model of DSAC remains to be tested, especially in the unique context of digital transformation of China’s service industry; second, the underlying mechanism of how DSAC translates into actual performance is still in a “black box” and has not yet been fully revealed. Second, the underlying mechanism of how DSAC translates into actual performance is still in a “black box” and has not yet been fully revealed.

Although existing studies have pointed out the importance of adaptive capability (AC) and customer-linking capability (CLC) in service competition [8,9], and found that data analytics can enhance both, whether there is an intrinsic link between AC and CLC, and whether DSAC can promote the synergistic development of the two are still unanswered questions. questions remain unanswered to date.

Based on the above judgment, this study introduces AC and CLC as mediating variables, selects a sample of 338 service firms in China that have implemented DSAC, and systematically explores the following two core questions with the help of structural equation modeling: (1) Is the conceptual structure of DSAC applicable to oriental cultural contexts? (2) Do adaptive capability (AC) and customer-linking capability (CLC) constitute a chain mediating path for DSAC to influence service performance? Through a series of rigorous empirical analyses, we validate for the first time the applicability of DSAC in the Chinese cultural context and construct a complete chain mediation model of “DSAC→AC→CLC→FP”.

The structure of this paper is organized as follows. Following the introduction, the Literature Background section reviews the theoretical development of dynamic capabilities and DSAC, laying the foundation for the study. Next, the Research Hypotheses section elaborates on the six hypotheses concerning DSAC, AC, CLC, and FP. The Research Methodology section then explains the applicability of Partial Least Squares Structural Equation Modeling (PLS-SEM) and describes the hypothesis testing process. Subsequently, the Results section presents the findings on whether the hypotheses are supported, and the Main Findings section interprets these results. Finally, the Discussion section explains the theoretical and practical contributions, summarizes the study’s limitations, and suggests directions for future research.

The contribution of this study at the theoretical level is that it confirms the universality of the higher-order competency framework in the new cultural context and reveals the micro-transmission mechanism that transforms dynamic service analytic competencies into performance outcomes in the process of digital transformation. The practical contribution is that it provides an actionable tool for service companies to optimize their DSAC investments, and helps managers to identify bottlenecks in the intermediary path, enabling them to establish a framework that covers “service analytics”, “organizational adaptation” and “customer connection”. Closed-loop system covering “service analysis”, “organizational adaptation” and “customer connection”.

## Literature background

### From dynamic capabilities theory to dynamic service analytics capabilities

The general framework for this study is provided by the dynamic capabilities theory. The basic proposition of this theory is that in a rapidly changing environment, a firm’s sustained competitive advantage does not depend primarily on a combination of resources at a particular point in time, but more critically comes from a firm’s ability to continually integrate, structure, and reorganize its resources [10]. In terms of process, this dynamic process is often described as a cycle of “sensing” market opportunities, ‘seizing’ them, and “reorganizing” corporate assets [11].

The situation is further different in the fast-paced, experience-heavy service industry environment. Due to high competitive pressure, real-time perception of subtle changes in customer needs becomes particularly important, and it is clear that service firms need a finer-grained, dynamic capabilities to guide their operations, and it is this real-world context that has given rise to the proposal of DSAC [4]. According to Akter et al.‘s understanding, DSAC focuses on real-time analysis of service elements and dynamic perception of market changes, which can essentially be regarded as the embodiment of the “perception” dimension of dynamic capabilities in service scenarios. However, it is puzzling that despite the forward-looking nature of the DSAC concept, academic progress has been surprisingly slow since its inception, and the research topics around DSAC proposed by Akter and his team in 2018 and 2020 have not been pursued, making DSAC almost a theoretically shelved tool. Interestingly, in stark contrast to the theoretical stagnation, there has been a rapid spread of dynamic service analytics in practice, whether it is real-time patient flow optimization in healthcare services [12], personalized recommendations with response times of less than 500 milliseconds in e-commerce services [13], or dynamic risk assessment in fintech services [14], and in practice, numerous companies are already quietly investing in the DSAC described by Those core competencies described by DSAC are already being benefited by many organizations in practice.

Therefore, it is the recovery and extension of the DSAC framework at the empirical level that this study aims to address. Not only do we want to verify the applicability of DSAC in other cultural contexts, but we also want to delve into the “black box” impact of DSAC: that is, the specific intermediary mechanisms through which these capabilities, which is so powerful in practice and so theoretically stagnant, ultimately affects the performance of service firms.

### Adaptive capability

Adaptive capability (AC) has long been understood as an organization’s ability to coordinate internal resources to match external demands in response to environmental changes [15]. AC is naturally coupled with the “sense-making” function of DSAC, which translates data-driven insights into operationally meaningful organizational change. Most existing studies recognize AC as a key mediator connecting strategic resources and performance outcomes, thus demonstrating the importance of AC as a mediator [8,16–18]. However, little of the current discussion on AC has been systematically embedded in the DSAC framework. Can AC be a mediating pathway for translating DSAC into performance? This potential issue deserves further clarification. In addition, the question of how AC affects other CLC in a DSAC-driven context also remains unexplored.

### Customer-linking capability

Customer-linking capability (CLC), based on CRM theory, is the ability of an organization to build and maintain strong customer relationships [19], and prior research has demonstrated CLC as a key driver of performance [20,21]. In the digital era, the expectation of instant service has brought the importance of CLC to an unprecedented level [22]. Also existing studies have shown that perceptual capabilities based on big data analytics [23], digital marketing capabilities [24], and strategic synergies of big data analytics initiatives [25] significantly enhance CLC, which can be seen as a driver of data analytics for CLC. However, existing studies have not yet placed CLC in a real-time dynamic environment to test whether it serves as a mediating mechanism for DSAC to influence FP. In addition, the potential interaction between AC and CLC has not been explored, which becomes a key missing link in understanding how data-driven adaptation translates into market success.

## Research hypothesis

### Dynamic service analysis capabilities and their impact on firm performance

Dynamic capabilities theory posits that in rapidly changing environments, companies possessing robust dynamic capabilities can achieve superior performance by continuously sensing external changes, capturing key information, and reconfiguring their resources [26,27]. Akter et al. argue that DSAC, as a domain-specific dynamic capabilities, enables service companies to analyze business environments, predict trends, and make informed decisions in near real-time. Previous research by Akter et al. confirmed that DSAC enhances performance by increasing organizational agility [3,4]. Within China’s highly dynamic digital markets—such as e-commerce, logistics, and fintech—intensifying competition and the growing importance of customer experience as a competitive advantage are driving organizations to invest in rapid service analytics [28,29]. Consequently, DSAC’s role may become even more critical. Based on the discussion, this study proposes the following hypothesis.

H1: DSAC positively influences FP in China’s digital service context

### The mediating role of adaptive capability

DSAC’s ability to generate real-time insights based on consumer big data that reflect changes in the marketplace has been shown to have a direct positive impact on firm performance (FP). However, this is not the only way to realize its value, and the effectiveness of DSAC may largely depend on a key intermediary mechanism, the organization’s adaptive capability (AC), which translates insights into actionable operational measures [18]. AC is defined as the ability of an organization to reconfigure its operational processes and resource allocations based on insights [30], and in China’s fast-changing economy, the ability of DSAC to generate real-time insights based on consumer big data has been proven to have a direct positive impact on FP ([31]), which is particularly critical in China’s fast-changing digital marketplace. A typical example is that at the beginning of the COVID-19 pandemic, JD.com Logistics found a significant rise in the number of masks in Wuhan through data monitoring, and then based on this information, stocked masks for Wuhan couriers, as well as conducted rapid replenishment and internal alerts [32].The correlation between adaptive capability and performance has also been demonstrated to a considerable extent in the Chinese context [33].

DSAC insights into customer needs (e.g., the need to capture those subtle personalized recommendations [34]) enable firms to implement real-time, high-quality adaptations for their customers, and often such adaptations promote trust building between firms and their customers [35], and given that trust is a core driver of customer loyalty [36,37], it may be prudent to infer that these insight-driven adaptations are ultimately more likely to strengthen the firm-customer link. Therefore, the following hypotheses are proposed:

H2: DSAC significantly influences FP through AC.
H3: DSAC significantly influences CLC through AC.

### The mediating role of a customer-linking capability

DSAC enables Chinese service firms to capture customer data in real time across a variety of contexts and identify unmet needs. This deep understanding is beneficial to service firms because they can build and maintain long-term customer relationships by creating contextualized interactions [38]. In a relationship-oriented market such as China, CLC directly drives FP because strong customer relationships enhance repeat purchase rates and word-of-mouth referrals, both of which are critical to growing market share and gaining performance [39]. One of the most prominent examples is the high performance of a Chinese fintech application service company that has cultivated a large group of loyal customers through full-cycle service accompaniment.

Adaptability (AC) enables companies to adjust processes, but these adjustments can only be translated into performance (FP) if they are communicated and validated through customer contact (CLC). For example, Chinese e-commerce firms use AC to optimize their return policies, and it is only through personalized messages that CLC can ensure that customers understand the policies, thus reducing complaints and increasing satisfaction [40]. Established research confirms that consumers in Eastern contexts value relational trust more than just functional adjustment [41], and thus CLC may play a mediating role between AC and FP. Therefore, the following hypotheses are proposed:

- H4: DSAC significantly influences FP through CLC.
- H5: AC significantly influences FP through CLC.

### Chain mediating role of adaptive capability and customer-linking capability

Service organizations use DSAC to sense key changes in market and customer needs, and then use AC to translate these changes into operational-level adjustments, such as improving service processes or updating content delivery. It is worth noting that some modern service firms have leveraged artificial intelligence tools to make this adjustment process real-time and adaptive. Taking adaptive recommender systems as an example [42], most online platform recommender systems can recommend services of interest to customers in real time based on changes in user preferences. Similarly, adaptive educational services [43], adaptive bit rate adjustment for web video services [44], adaptive navigation for maps, adaptive fitness programs for health-focused apps and adaptive AI customer service for customer service platforms are facts that reflect the process from DSAC to AC.

Under the Service-Dominant Logic any adaptive behavior of the enterprise does not create value directly, but its value must be “determined” by the customer’s perceptions and interactions [45], therefore, the improvement of AC should ultimately affect the performance through the enhancement of CLC. Specifically, when a service firm responds quickly to customer needs through AC (e.g., real-time adjustment of services or personalization of content), it conveys a high degree of “caring” to the customer, and such positive interactions may significantly enhance the customer’s sense of trust and the quality of the relationship, which strengthens the link between the firm and the customer, a phenomenon which is not only a positive influence on the performance of the service firm, but also on the performance of the service firm in terms of “relationship”. This phenomenon is particularly salient in cultural contexts where “relationships” are important social capital. According to CRM, strong CLC is the cornerstone of business performance, and strong customer relationships mean higher customer loyalty, repurchase rates, and broader word-of-mouth referrals, which translate directly into superior performance. Therefore, the following hypotheses are proposed:

H6: DSAC significantly affects FP through the sequential mediating effects of AC and CLC.

### Conceptual model

**Fig 1:**
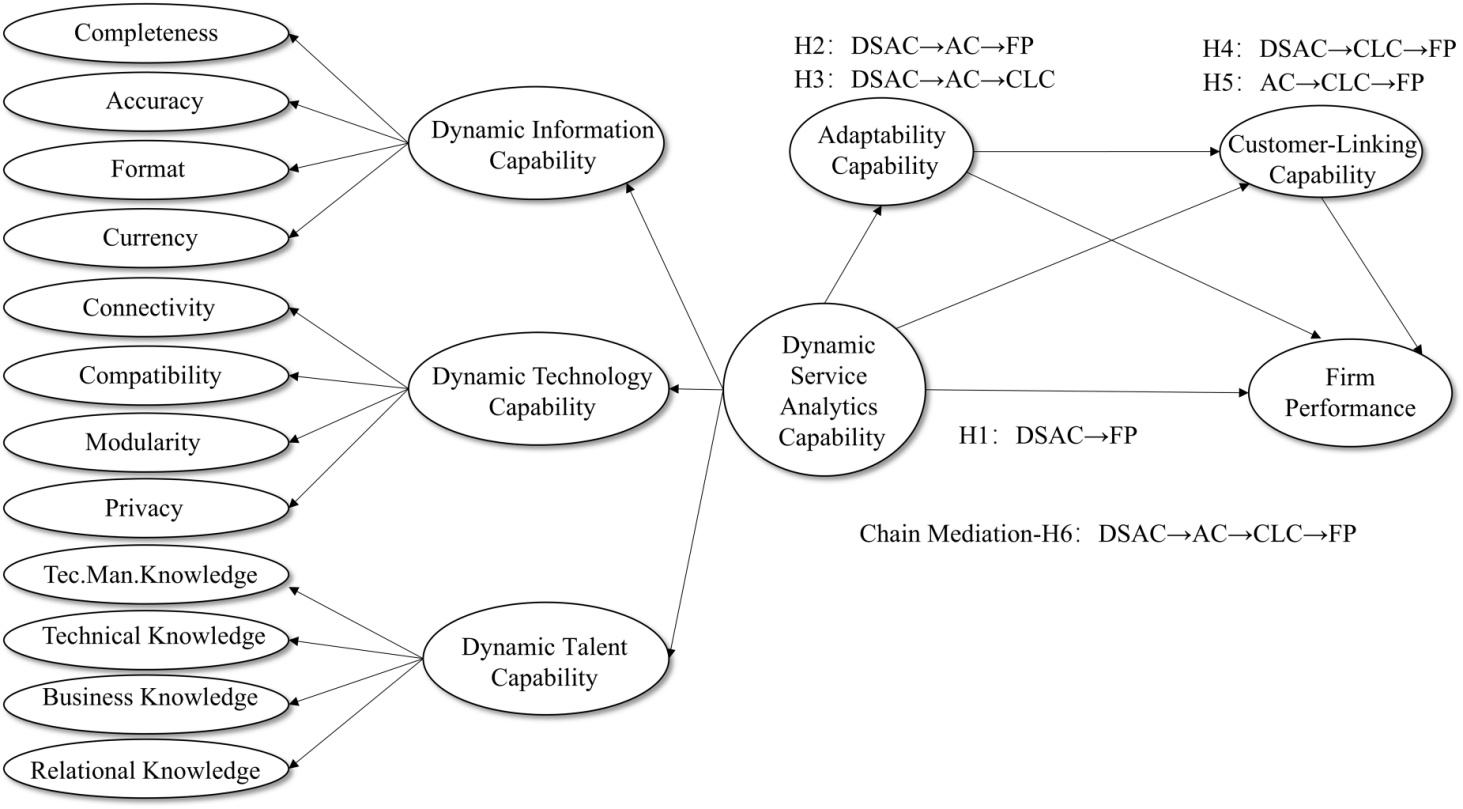
Conceptual model.

The central claim of this study is that the mechanism by which DSAC affects FP may be more convoluted than a direct causal association-it actually weaves through a synergistic mediating system consisting of AC and CLC. The model in Fig 1 outlines three possible pathways. DSAC has a direct enhancement on FP which is the starting point. Furthermore, it also acts indirectly on FP by enhancing AC and optimizing CLC, respectively, but the key here lies in the third path: AC and CLC are not isolated from each other, but form a coherent conduction sequence (DSAC → AC → CLC → FP). In other words, with DSAC, firms increase their internal resilience, which in turn makes them more capable of fostering customer-centered external linking capabilities that ultimately lead to performance. In this way, this chain model may be a useful extension of the traditional intermediation framework, which attempts to reveal that the realization of the value of technology investments actually follows a complete trajectory from “intra-organizational adaptation” to “market-side linkage”.

## Research methodology

### Scale development

The measurement instrument was mainly based on the reliability-tested scales from established studies (see Appendix 1 for the complete items). To ensure cross-cultural validity, we implemented a rigorous translation and back-translation process for the original English questionnaire: first, the Chinese forward translation was completed by a bilingual expert, followed by the English back-translation by an independent translator, and finally, semantic and contextual differences were resolved through multiple rounds of comparison and discussion involving data analysts from three Chinese data service companies.

On this basis, we further organized a pre-test with a sample of 100 middle managers in the finance, logistics and retail industries. All participants were purposively sampled based on two criteria: first, they have no less than three years of work experience in their respective companies, and second, they have been directly involved in DSAC-related decision making. It is worth stating that the feedback from this pretest played a key role in the optimization of the scale, specifically in the improvement of item presentation clarity, contextual relevance and response format.

In terms of measurement, all constructs were measured on a 7-point Likert scale (1 = “strongly disagree”, 7 = “strongly agree”), which was designed to balance measurement efficiency and response consistency.

### Survey

The data collection of this study relies on the Chinese research platform Credamo.com, which covers about 3 million users and provides the basis for the broad representativeness of the sample. The data collection period is from May 17 to November 6, 2023, and the study targets middle- and senior-level professionals in enterprises that have deployed real-time service analytics systems, and all data are collected in the form of anonymous questionnaires. On the ethical level, the project was ethically reviewed by the School of Tourism and Sports of Hezhou University (Project No. 2023005), and written informed consent was obtained from all participants.

To ensure the validity of the study, we set three core entry criteria for respondents: (1) technical literacy in interpreting real-time analytics outputs; (2) authority to deploy resources related to optimizing DSAC; (3) no less than three years of experience in an enterprise that has implemented DSAC. We believe that these three conditions, while seemingly stringent, are necessary prerequisites for accurately capturing the subtle correlation between competence and performance.

The study sample covers a wide range of service industries, with the following composition: information transmission, software and information technology services (34.06%), scientific research and technology services (6.5%), financial services (16.27%), tourism, accommodation and food services (16.57%), wholesale and retail (7.1%), transportation, warehousing and postal services (7.4%), leasing and business services (7.1%), and residential services (5%). These companies were included in the study in large part because they have all realized visible operational improvements through real-time data analytics, making them ideal candidates for our exploration of this topic. In terms of geographic distribution, the respondents cover 27 provinces and cities in China, with Shanghai, Guangdong, Zhejiang, Jiangsu and other economically active regions especially represented, which to some extent also enhances the regional representativeness of the sample.

In the data cleaning stage, this study initially excluded about 30% of invalid questionnaires (N = 460) by using the built-in mechanism of Credamo.com, based on the following behaviors: completion time less than 150 seconds, contradictory answers to reverse questions, and mechanical linear responses. Based on this, the manual review was further supplemented to exclude another 30 responses with logical inconsistencies. After such rounds of cleaning, the final number of valid samples retained was 338, with an effective recovery rate of 73.5%.

### Data analysis

In this study, a reflective-reflective structure was used in the measurement model, and all first-, second-, and third-order constructs were modeled with reflective metrics. The DSAC was considered a higher-order constructs, which contains 12 dimensions in the first-order constructs, 4 dimensions in the second-order constructs, and 3 dimensions in the third-order constructs. In terms of method selection, Partial Least Squares Structural Equation Modeling (PLS-SEM) is used in this study for the following reasons:(1) Because DSAC is a complex hierarchical constructs, PLS-SEM is able to better estimate the complex hierarchical constituent model through techniques such as the repetition indicator method, whereas CB-SEM is susceptible to model identification problems [46]. (2) In this study its dual purpose of both exploratory and predictive, exploring chain mediation models with complex constructs involved, in which PLS-SEM demonstrates a unique advantage [47]. (3) PLS-SEM prioritizes variance interpretation and predictive power over model fit, which is consistent with our goal [48]. (4) Given the limited sample size (N = 338) and the need to reduce model complexity, PLS-SEM does have a better tolerance for small samples and non-normal distributions of the data relative to CB-SEM, which is suitable for dealing with complex models and sample sizes that are not very large [47]. (5) The method has been widely recognized in the field of data analysis capabilities for assessing complex models [49,50]. In this study, the bootstrap method with 5000 iterations was used to estimate the standard errors. In addition, the repeated indicator method with path weighting scheme was used to determine the optimal model parameters. Following the hierarchical component modeling methodology, lower-order constructs were always used to calculate higher-order construct scores. Accordingly, DSAC represents the aggregation of all corresponding indicators under its first-order latent variables. In the specific estimation process, we use the Bootstrap method with 5000 repeated samples to calculate the standard errors, and at the same time, we combine the repeated indicator method with the path weighting scheme in order to determine the optimal model parameters [51]. Following the typical logic of hierarchical component model construction, the scores of all higher-order constructs (including DSAC) are calculated based on their subordinate lower-order constructs. Therefore, the DSAC reported in this study essentially represents a systematic aggregation of all the indicators corresponding to its next order latent variables.

### Common method bias

In questionnaire studies, common method bias (CMB) may arise when a single respondent completes all items. To mitigate this potential effect, the study included reverse coded items at the questionnaire design stage (e.g., reverse items were set in the initial “culture of analysis” construct, although this construct was excluded from the final model in subsequent analyses), which helped to identify possible habitual patterns of respondent answering, thereby reducing false correlation generation. The study also performed a Harman’s one-way test by analyzing the unrotated exploratory factor analysis. The results showed that: the first principal component explained 34.791% of the variance, a value below the recognized empirical threshold of 40% [52]. Further, the study followed the recommendation of [53] to implement a full covariance test, and the variance inflation factor (VIF) for all constructs ranged from 1.000 to 1.836, which is well below the commonly used threshold value of 3.3. Taken together, the results of these tests give reason to believe that common method bias does not appear to pose a serious problem in this study.

### Measurement model

The preliminary evaluation of the measurement model involved assessing the factor loadings, composite reliability  (CR), and average variance extracted  (AVE) for the first-order constructs, as presented in Table 1. According to standard guidelines for PLS modeling, acceptable thresholds require item loadings greater than 0.70, CR values above 0.70, and AVE values exceeding 0.50 [48]. In this study, all item loadings exceeded 0.80, indicating strong convergent validity when measuring their respective constructs. Additionally, both CR and AVE  values surpassed their recommended thresholds, thus confirming the reliability and internal consistency of the measurement scale.

**Table 1:** Measurement model.

Dimensions	Sub-dimensions	Items	Loading	CR	AVE
Dynamic information capability	Completeness (COM)	COM1	0.882	0.861	0.782
		COM2	0.885		
		COM3	0.886		
	Currency (CUR)	CUR1	0.875	0.883	0.740
		CUR2	0.856		
		CUR3	0.862		
		CUR4	0.846		
	Format(FOR)	FOR1	0.889	0.852	0.770
		FOR2	0.880		
		FOR3	0.863		
	Accuracy (ACC)	ACC1	0.885	0.865	0.787
		ACC2	0.890		
		ACC3	0.887		
Dynamic technology capability	Connectivity (CON)	CON1	0.886	0.862	0.784
		CON2	0.884		
		CON3	0.886		
	Compatibility (COMP)	COMP1	0.887	0.864	0.786
		COMP2	0.892		
		COMP3	0.881		
	Modularity (MOD)	MOD1	0.866	0.862	0.783
		MOD2	0.897		
		MOD3	0.891		
	Privacy (PRI)	PRI1	0.876	0.865	0.787
		PRI2	0.898		
		PRI3	0.888		
Dynamic talent capability	Technical knowledge (TK)	TK1	0.840	0.874	0.725
		TK2	0.839		
		TK3	0.848		
		TK4	0.877		
	Technical management knowledge (TM)	TM1	0.865	0.887	0.746
		TM2	0.868		
		TM3	0.859		
		TM4	0.863		
	Business knowledge (BK)	BK1	0.867	0.891	0.754
		BK2	0.866		
		BK3	0.878		
		BK4	0.861		
	Relational knowledge(RK)	RK1	0.850	0.885	0.742
		RK2	0.852		
		RK3	0.872		
		RK4	0.870		
Adaptive capability (AC)		AC1	0.867	0.925	0.746
		AC2	0.842		
		AC3	0.840		
		AC4	0.854		
		AC5	0.857		
		AC6	0.852		
Customer- linking capability(CLC)		CLC1	0.892	0.865	0.787
		CLC2	0.884		
		CLC3	0.885		
Firm performance (FP)		FP1	0.861	0.872	0.722
		FP2	0.828		
		FP3	0.875		
		FP4	0.834		

The correlation matrix in Table 2 confirms discriminant validity by displaying the square roots of the AVE values along the diagonal, which are higher than the intercorrelations between the first-order constructs [54]. This test supports the theoretical distinctiveness of the constructs, indicating that they measure different concepts and are not redundant. Additionally, cross-loading analysis further validates this finding by demonstrating that each item loads more strongly on its corresponding construct than on any other construct. The first-order measurement model demonstrated robustness, as evidenced by its satisfactory reliability, convergent validity, and discriminant validity. Consequently, the measurement model was deemed adequate, providing a solid foundation for subsequent research.

**Table 2:** Correlations and average variance extracted.

Construct	COM	CUR	FOR	ACC	CON	COMP	MOD	PRI	TK	TM	BK	RK	SA	CLC	FP
Completeness (COM)	0.884														
Currency (CUR)	0.356	0.860													
Format (FOR)	0.454	0.356	0.878												
Accuracy(ACC)	0.408	0.338	0.458	0.887											
Connectivity (CON)	0.421	0.300	0.437	0.433	0.885										
Compatibility (COMP)	0.389	0.326	0.473	0.487	0.456	0.886									
Modularity (MOD)	0.374	0.248	0.438	0.446	0.505	0.481	0.885								
Privacy (PRI)	0.441	0.282	0.467	0.430	0.502	0.479	0.456	0.887							
Technical knowledge (TK)	0.387	0.260	0.449	0.403	0.432	0.399	0.387	0.417	0.851						
Technical management knowledge (TM)	0.419	0.330	0.527	0.416	0.425	0.473	0.418	0.458	0.420	0.864					
Business knowledge (BK)	0.450	0.388	0.405	0.403	0.423	0.456	0.407	0.443	0.376	0.443	0.868				
Relational knowledge (RK)	0.503	0.346	0.465	0.415	0.522	0.465	0.471	0.510	0.448	0.472	0.466	0.861			
Adaptive Capability (AC)	0.347	0.302	0.409	0.478	0.410	0.384	0.390	0.418	0.405	0.419	0.391	0.390	0.852		
Customer-Linking Capability (CLC)	0.293	0.229	0.371	0.398	0.300	0.329	0.272	0.323	0.320	0.309	0.253	0.338	0.574	0.887	
Firm performance (FP)	0.320	0.272	0.403	0.372	0.304	0.378	0.297	0.350	0.356	0.309	0.315	0.367	0.545	0.606	0.850

### Higher-order measurement model

Considering the hierarchical structure of the conceptual model, this study evaluates the measurement attributes of the higher-order constructs, specifically the third-order DSAC and the second-order constructs: dynamic information capability, dynamic technology capability, and dynamic talent capability, as detailed in Table 3. The third-order DSAC comprises 41 items, including 13 items for dynamic information capability, 12 items for dynamic technology capability, and 16 items for dynamic talent capability. As the DSAC was modeled reflectively, this study confirmed that the higher-order path coefficients were statistically significant. For instance, dynamic information capability accounts for approximately 86% of the variance, dynamic technology capability explains 88%, and dynamic talent capability explains 93% of the variance. As shown in Table 4, dynamic information capability is primarily reflected in information completeness (74%), currency (71%), format (76%), and accuracy (74%). Dynamic technology capability is measured through connectivity (79%), compatibility (77%), modularity (78%), and privacy (78%). Dynamic talent capability is explained by technical knowledge (72%), technical management knowledge (77%), business knowledge (75%), and relational knowledge (79%). These indicators are statistically significant at P<0.001.

**Table 3:** Evaluation of the higher-order measurement model.

Models	Latent constructs	AVE	CR	Dimensions	β	t-statistic	
Third-order	Dynamic Service Analytics Capabilities (DSAC)	0.814	0.929	INMAC	0.858	53.621	
				TENOC	0.875	68.219	
				TALEC	0.926	93.659	
**Models**	**Latent constructs**	**AVE**	**CR**	**Dimensions**	**β**	**R square**	**t-statistic**
Second-order	Dynamic Information Capability (INMAC)	0.546	0.828	COM	0.743	0.552	20.140
				CUR	0.711	0.506	20.280
				FOR	0.763	0.582	26.812
				ACC	0.739	0.546	25.918
	Dynamic Technology Capability (TENOC)	0.610	0.862	CON	0.790	0.624	31.084
				COMP	0.772	0.596	29.589
				MOD	0.780	0.608	33.022
				PRI	0.781	0.611	33.345
	Dynamic Talent Capability (TALEC)	0.579	0.846	TK	0.724	0.524	18.965
				TM	0.771	0.595	25.721
				BK	0.753	0.567	23.281
				RK	0.793	0.628	30.076

**Table 4:** Results of structural model analysis.

Hypotheses	Main model	Path coefficients	Boot SEs	t-statistic	p	LLCI	ULCI	VAF
H1	DSAC→FP	0.191	0.072	2.638	0.008	0.048	0.329	
	**Interaction model**							
H2	DSAC→AC → FP	0.118	0.041	2.700	0.007	0.037	0.208	24%
H3	DSAC→AC → CLC	0.270	0.043	6.264	0.000	0.185	0.353	59.6%
H4	DSAC→CLC → FP	0.074	0.033	2.218	0.027	0.013	0.145	15%
H5	AC → CLC → FP	0.188	0.041	4.609	0.000	0.111	0.272	47.8%
H6	DSAC→AC → CLC → FP	0.109	0.024	4.463	0.000	0.064	0.160	22.2%

### Structural model

The research developed a structural model to estimate the hypothesized relationships among DSAC, AC, CLC, and FP. Table 4 presents the key path coefficients of the main model. The study confirms Hypothesis 1 (H1) by demonstrating that higher-order DSAC significantly affects FP. Furthermore, the study investigates the interaction model, examining AC’s mediating role between DSAC and both FP and CLC. The mediating effects of CLC were also analyzed, Specifically, its role between DSAC and FP, as well as between AC and FP. The results confirmed that the mediating roles of AC and CLC were statistically significant, with t-values exceeding 1.96  (p < 0.05), thereby supporting Hypotheses 2, 3, 4, and 5. Additionally, this study validates Hypothesis 6 (H6) by confirming the chain mediation effect of AC and CLC in the relationship between DSAC and FP.To assess the mediation effects, this study employed VAF values, which are calculated as the ratio of the indirect effect to the total effect [48]. Path analysis revealed a chain mediation pattern: approximately 24% of DSAC’s effect on FP is mediated through AC, and 15% through CLC. Furthermore, AC partially mediates the relationship between DSAC and CLC, accounting for 60% of this effect. CLC also partially mediates the relationship between AC and FP, accounting for 48% of the effect. Approximately 22% of DSAC’s influence on FP is attributed to the combined effects of both mediators. These findings indicate that AC and CLC jointly and partially mediate the DSAC-FP relationship, with each playing a distinct and complementary role. Additionally, the R² values for AC, CLC, and FP were 0.332, 0.352, and 0.449, respectively.

## Results

This study reveals that dynamic service analysis capabilities (DSAC) has a significant positive direct effect (p < 0.05) on firm performance (FP) and hypothesis H1 is supported. This finding suggests that service-oriented firms can improve their overall performance by developing and effectively utilizing DSAC. Adaptive capability (AC) partially mediates the relationship between DSAC and FP (VAF = 24%), validating hypothesis H2. similarly, AC partially mediates the relationship between DSAC and customer-linking capability (CLC) (VAF = 60%), supporting hypothesis H3. these findings suggest that AC is not only a critical pathway through which DSAC affects performance, but also a core enhancement of the CLC mechanism.CLC has a mediating effect in the relationship between DSAC and FP (p<0.05),supporting hypothesis H4.In addition, CLC plays a partial mediating role between customer affiliation and financial performance (VAF=48%), empirically supporting hypothesis H5.This study also validates the chain mediation path of DSAC→AC → CLC → FP, which presents a partial mediating effect ( VAF≈22%), supporting hypothesis H6.This serial mediation mechanism suggests that DSAC enhances firm performance by progressively strengthening AC and CLC, revealing the intrinsic operating mechanism of multi-stage capabilities development.

## Main findings

This study validates the higher-order conceptual structure of DSAC in an eastern cultural context, and the results show that the factor loadings of all dimensions perform satisfactorily with the fit of the measurement model, which suggests that the DSAC framework, which is initially rooted in a western context, has equally good explanatory power when applied to eastern service firms. In this sense, this study expands the application boundaries of the theoretical framework to some extent.

In addition, the study confirms the existence of a direct contribution of DSAC to service FP, and also finds that AC plays a key mediating role between DSAC and performance, which prompts us to revisit the value realization mechanism of DSAC: it does not act directly on performance, but needs to be transformed through adaptive mechanisms within the organization. Moreover, the mediating effect of AC between DSAC and CLC reveals the pivotal function it plays between data and customer relationships. The mediating effect of CLC between DSAC and FP further suggests that service firms may be able to use real-time data analytics to enhance the depth of customer relationships and thus improve overall performance. The study also confirms that CLC exhibits a clear mediating effect between AC and FP. This finding suggests that an organization’s adaptive capability also needs to be transformed into performance outcomes ultimately through strong customer connections, which is very much in line with the characteristics of the cultural context of China’s relational society.

Summarizing these findings, the core theoretical contribution of this study is the identification of a chain mediation path of DSAC mediated through AC to CLC, which ultimately affects FP. This path not only presents the multi-stage and progressive characteristics of DSAC’s influence on performance, but also outlines the evolutionary logic of “data-adaptation-relationship-performance”. It can be said that this reflects the gradual process from “data empowerment” to “organizational adaptation” to “customer empathy” in the practice of digitalization process of service enterprises.

## Discussion

Based on an empirical analysis of 338 service firms in China, this study confirms the applicability of the DSAC proposed by Akter et al [3,4] in an Eastern cultural context. This finding responds to the growing call within the field of business capabilities research that new theoretical concepts of capabilities need to be urgently tested and scrutinized in multicultural contexts [55,56]. In addition, while earlier literature [3] established a direct link between DSAC and performance, our study takes previous research a step further by revealing the mediating mechanism of the “DSAC→AC→CLC→FP” chain, which attempts to pry open the “black box” of how DSAC creates performance. “black box””. This finding is highly compatible with the core proposition of dynamic capabilities theory that “capabilities are transformed through mediating mechanisms” [10], and at the same time, further refines and extends the conduction path of the theory under the domain of digital services.

### Theoretical contributions

The theoretical contributions of this study are mainly in two aspects. First, by empirically testing the higher-order structure of DSAC in an Eastern cultural context, the cross-cultural robustness and theoretical generalizability of the concept are enhanced. This is not simply a repetitive validation, but a conscious expansion of the research landscape of dynamic capabilities in service industries. Second, the chain mediation model “DSAC→AC→CLC→FP” is constructed and validated, which systematically reveals the micro-mechanism by which dynamic service analytic capabilities are ultimately transformed into firm performance. This path not only echoes the core principle of dynamic capabilities theory, “capabilities are transformed through intermediary mechanisms” [10], but also poses a challenge to the mainstream perspective of [8,25], which tends to examine the roles of AC and CLC in isolation. It is worth pointing out that our model reveals the synergistic and sequential effects of the two, which enriches the theoretical connotation of DSAC at a deeper level, and expands our understanding of the path of “capabilities-performance” transformation in the context of digital services.

### Managerial contributions

This study provides specific guidance for managers of service companies. First, managers should systematically invest in DSAC construction and strengthen the monitoring of the development status of AC and CLC on the path during practice. The path can be used to diagnose the bottleneck points encountered in the process of capabilities transformation, so as to optimize the allocation of resources. Second, managers should promote the in-depth integration of DSAC with organizational processes and customer management system to ensure that the results of data analysis can be quickly transformed into adaptive actions and accurate customer interactions. Finally, industry associations and government agencies can set standards and incentives to encourage service companies to invest in DSAC infrastructure and talent development, and work together to build a dynamic data-driven service ecosystem.

### Limitations and future directions of this research

Like other similar studies, this study also has some limitations, firstly, the sample focuses on Chinese service firms and the generalizability of the findings to other cultures or industries needs to be further verified. Secondly, the cross-sectional data is difficult to capture the dynamic evolution of the variables, and future research could adopt a longitudinal design to track the trajectory of DSAC formation. In addition, other potential mediating variables (e.g., digital leadership, adaptive innovation) or moderating variables (e.g., environmental uncertainty) can be explored to improve the theoretical model; finally, due to the rapid development of AI, what changes in the dimensions of the DSAC framework (e.g., information competence, technological competence, and talent competence) are generated by AI is a direction worthy of further investigation.

## Supporting information

S1 File Questionnaire information. (DOCX)

S1 Data Research data. (XLSX)

## References

[1] Kumar H. Enablers for digital transformation of services to harness new business opportunities. IEEE Trans Eng Manage. 2024;71:14282–92. doi: 10.1109/tem.2024.3437733

[2] Pitelis CN, Teece DJ, Yang H. Dynamic capabilities and MNE global strategy: a systematic literature review‐based novel conceptual framework. J Manag Stud. 2023. doi: 10.1111/joms.13021

[3] Akter S, Fosso Wamba S, Barrett M, Biswas K. How talent capability can shape service analytics capability in the big data environment? J Strat Mark. 2018;27(6):521–39. doi: 10.1080/0965254x.2018.1442364

[4] Akter S, Motamarri S, Hani U, Shams R, Fernando M, Mohiuddin Babu M, et al. Building dynamic service analytics capabilities for the digital marketplace. J Bus Res. 2020;118:177–88. doi: 10.1016/j.jbusres.2020.06.016

[5] Cabrera Jojoa CH, Svorobej S, Palade A, Kazmi A, Clarke S. MAACO: a dynamic service placement model for smart cities. IEEE Trans Serv Comput. 2022:1–1. doi: 10.1109/tsc.2022.3143029

[6] Khurana S, Deb N, Mistry S, Ghose A, Krishna A, Dam HK. Egalitarian transient service composition in crowdsourced IoT environment. IEEE Trans Serv Comput. 2023;16(5):3305–17. doi: 10.1109/tsc.2023.3264581

[7] Sathupadi K, Achar S, Bhaskaran SV, Faruqui N, Uddin J. BankNet: real-time big data analytics for secure internet banking. BDCC. 2025;9(2):24. doi: 10.3390/bdcc9020024

[8] Uzkurt C, Ekmekcioglu EB, Ceyhan S. Business ties, adaptive capability and technological turbulence: implications for SMEs’ performance in Turkey. JBIM. 2023;39(3):568–80. doi: 10.1108/jbim-01-2023-0049

[9] Eichholz J, Hoffmann N, Schwering A. The role of risk management orientation and the planning function of budgeting in enhancing organizational resilience and its effect on competitive advantages during times of crises. J Manag Control. 2024;35(1):17–58. doi: 10.1007/s00187-024-00371-8

[10] Teece DJ. Explicating dynamic capabilities: the nature and microfoundations of (sustainable) enterprise performance. SMJ. 2007;28(13):1319–50. doi: 10.1002/smj.640

[11] Teece DJ. The foundations of enterprise performance: dynamic and ordinary capabilities in an (economic) theory of firms. AMP. 2014;28(4):328–52. doi: 10.5465/amp.2013.0116

[12] Li B, Li G, Liu J, Sun H, Wen C, Yang Y, et al. Deep-learning-based real-time individualization for reduce-order haemodynamic model. Comput Biol Med. 2024;174:108476. doi: 10.1016/j.compbiomed.2024.108476 38636328

[13] Li W, Cai Y, Hanafiah MH, Liao Z. An empirical study on personalized product recommendation based on cross-border E-commerce customer data analysis. J Organ End User Comput. 2024;36(1):1–16. doi: 10.4018/joeuc.335498

[14] Andronie M, Blažek R, Iatagan M, Skypalova R, Uță C, Dijmărescu A, et al. Generative artificial intelligence algorithms in Internet of Things blockchain-based fintech management. OC. 2024;15(4):1349–81. doi: 10.24136/oc.3283

[15] Keskin H, Akgün AE, Esen E, Yilmaz T. The manufacturing adaptive capabilities of firms: the role of technology, market and management systems-related adaptive capabilities. JMTM. 2022;33(8):1429–49. doi: 10.1108/jmtm-01-2022-0021

[16] Arraya M. Distinctive capabilities system in MSME’s business model adaptation: evidence of the moderating and mediating effect of adaptive capability. Centr Europ Manag J. 2024;33(2):183–98. doi: 10.1108/cemj-11-2023-0438

[17] Rangaswamy US, Chaudhary S. Effect of adaptive capability and entrepreneurial orientation on SBU performance: moderating role of success trap. MRR. 2021;45(3):436–49. doi: 10.1108/mrr-01-2021-0027

[18] Kuo S-Y. Improving innovation performance through learning capability and adaptive capability: the moderating role of big data analytics. Knowl Manag Res Pract. 2023;22(4):364–76. doi: 10.1080/14778238.2023.2212182

[19] Singh R, Charan P, Chattopadhyay M. Effect of relational capability on dynamic capability: exploring the role of competitive intensity and environmental uncertainty. J Manag Organ. 2022;28(3):659–80. doi: 10.1017/jmo.2022.27

[20] Hossain MA, Akter S, Yanamandram V. Why doesn’t our value creation payoff: Unpacking customer analytics-driven value creation capability to sustain competitive advantage. J Bus Res. 2021;131:287–96. doi: 10.1016/j.jbusres.2021.03.063

[21] Cao G, Weerawardena J. Strategic use of social media in marketing and financial performance: the B2B SME context. Ind Mark Manag. 2023;111:41–54. doi: 10.1016/j.indmarman.2023.03.007

[22] Ku ECS, Chen C-D. Increasing the organizational performance of online sellers: the powerful back-end management systems. BPMJ. 2023;29(3):838–57. doi: 10.1108/BPMJ-11-2022-0562

[23] Fosso Wamba S, Queiroz MM, Wu L, Sivarajah U. Big data analytics-enabled sensing capability and organizational outcomes: assessing the mediating effects of business analytics culture. Ann Oper Res. 2020;333(2–3):559–78. doi: 10.1007/s10479-020-03812-4

[24] Liu Y. Effect of digital marketing capabilities and blockchain technology on organizational performance and psychology. Front Psychol. 2022;12:805393. doi: 10.3389/fpsyg.2021.805393 35211056 PMC8862735

[25] Itani OS, Kalra A, Rostami A. How does big data affect organizational financial performance in turbulent markets? The role of customer-linking and selling capabilities. Technol Forecast Soc Change. 2024;201:123221. doi: 10.1016/j.techfore.2024.123221

[26] Jin X, Wu Y. How does digital transformation affect the ESG performance of Chinese manufacturing state-owned enterprises?-Based on the mediating mechanism of dynamic capabilities and the moderating mechanism of the institutional environment. PLoS One. 2024;19(5):e0301864. doi: 10.1371/journal.pone.0301864 38743669 PMC11093376

[27] Zhang L, Ye Y, Meng Z, Ma N, Wu C-H. Enterprise digital transformation, dynamic capabilities, and ESG performance. J Glob Inf Manag. 2024;32(1):1–20. doi: 10.4018/jgim.335905

[28] Li J, Bonn MA, Wang J, Cho M. Food delivery application user segmentation in the mobile marketing world in China. J Asia Pac Econ. 2021;28(2):484–501. doi: 10.1080/13547860.2021.1969839

[29] Liu X, Zhang N, Hao X. A Strategic model for service-oriented enterprises based on online reviews: the research of budget hotel chains in China. Inf Technol Manag. 2024;26(3):361–78. doi: 10.1007/s10799-024-00417-2

[30] Rangaswamy US, Batra S. “Can’t direct the wind? Adjust the sails”: a mediation model of intellectual capital, adaptive capability and project performance. JIC. 2025;26(2):362–79. doi: 10.1108/jic-04-2024-0102

[31] Akter S, Bandara RJ, Sajib S. How to empower analytics capability to tackle emergency situations? IJOPM. 2021;41(9):1469–94. doi: 10.1108/ijopm-11-2020-0805

[32] Shen ZM, Sun Y. Strengthening supply chain resilience during COVID‐19: a case study of JD.com. J Oper Manag. 2021;69(3):359–83. doi: 10.1002/joom.1161

[33] Zhu D. Research on the innovation performance of NEV enterprises driven by AI technology: an empirical study based on China’s NEV industry. K. 2025. doi: 10.1108/k-11-2024-3026

[34] Ding L, Antonucci G, Venditti M. Unveiling user responses to AI-powered personalised recommendations: a qualitative study of consumer engagement dynamics on Douyin. Qual Mark Res. 2024;28(2):234–55. doi: 10.1108/qmr-11-2023-0151

[35] Pichot L, Pierre J. Importance of informal skills for sporting goods retailers in the service relationship. Relat Ind. 2024;79(3–4). doi: 10.7202/1118803ar

[36] Orazgaliyeva E, Abuzhalitova A, Sokhatskaya N, Smykova M, Kazybayeva A. Trust as a critical driver of customer loyalty in the pharmaceutical market: a study of Kazakhstan. RSPP. 2024;16(9):100021. doi: 10.1016/j.rspp.2024.100021

[37] Van Huy L, Thinh NHT, Thi Thu Dung T, Duc Tien N. Trust, satisfaction and co-creation - an approach to corporate social responsibility in the hospitality industry: a roadmap towards customer loyalty. J Qual Assur Hosp Tour. 2025:1–26. doi: 10.1080/1528008x.2025.2467449

[38] Pandey PK, Pandey PK. Unveiling the transformative power of augmented reality in retail: a systematic literature analysis. J Strategy Manag. 2025. doi: 10.1108/jsma-05-2023-0101

[39] Liu X, Liu P, Li M. Factors influencing value co-creation in cultural and creative enterprises: an empirical study. Heliyon. 2024;10(15):e35100. doi: 10.1016/j.heliyon.2024.e35100 39170346 PMC11336428

[40] Zhou K, Zhang H, Fang Z, Qin Y. The impact of consumers’ strategic return behavior on review bias: evidence from Chinese online fashion retail. J Retail Consum Serv. 2026;88:104550. doi: 10.1016/j.jretconser.2025.104550

[41] Liu M, Xu J, Li S, Wei M. Engaging customers with online restaurant community through mutual disclosure amid the COVID-19 pandemic: the roles of customer trust and swift guanxi. J Hosp Tour Manag. 2023;56:124–34. doi: 10.1016/j.jhtm.2023.06.019

[42] Yuan Y, Zheng W. Your trip, your way: an adaptive tourism recommendation system. Appl Soft Comput. 2024;154:111330. doi: 10.1016/j.asoc.2024.111330

[43] Han J, Liu G, Liu X, Yang Y, Quan W, Chen Y. Continue using or gathering dust? A mixed method research on the factors influencing the continuous use intention for an AI-powered adaptive learning system for rural middle school students. Heliyon. 2024;10(12):e33251. doi: 10.1016/j.heliyon.2024.e33251 39022032 PMC11252867

[44] Yang H, Liu Q, Song C. Adaptive QP algorithm for depth range prediction and encoding output in virtual reality video encoding process. PLoS One. 2024;19(9):e0310904. doi: 10.1371/journal.pone.0310904 39321161 PMC11424003

[45] Jaakkola E, Kaartemo V, Siltaloppi J, Vargo SL. Advancing service-dominant logic with systems thinking. J Bus Res. 2024;177:114592. doi: 10.1016/j.jbusres.2024.114592

[46] F. Hair Jr J, Sarstedt M, Hopkins L, G. Kuppelwieser V. Partial least squares structural equation modeling (PLS-SEM). EBR. 2014;26(2):106–21. doi: 10.1108/ebr-10-2013-0128

[47] Sabol M, Hair J, Cepeda G, Roldán JL, Chong AYL. PLS-SEM in information systems: seizing the opportunity and marching ahead full speed to adopt methodological updates. IMDS. 2023;123(12):2997–3017. doi: 10.1108/imds-07-2023-0429

[48] Hair JF Jr, Ringle CM, Sarstedt M. Partial least squares structural equation modeling: rigorous applications, better results and higher acceptance. Long Range Plann. 2013;46(1–2):1–12. doi: 10.1016/j.lrp.2013.01.001

[49] Ongena G, Davids A. Big data analytics capability and governmental performance. IJEGR. 2023;19(1):1–18. doi: 10.4018/ijegr.321638

[50] Rahman MdA, Saha P, Belal HM, Hasan Ratul S, Graham G. Big data analytics capability and supply chain sustainability: analyzing the moderating role of green supply chain management practices. BIJ. 2024;33(2):417–43. doi: 10.1108/bij-10-2024-0852

[51] Becker J-M, Klein K, Wetzels M. Hierarchical latent variable models in PLS-SEM: guidelines for using reflective-formative type models. Long Range Plann. 2012;45(5–6):359–94. doi: 10.1016/j.lrp.2012.10.001

[52] Podsakoff PM, Organ DW. Self-reports in organizational research: problems and prospects. J Manage Organ. 1986;12(4):531–44.

[53] Kock N. Common method bias in PLS-SEM. IJeC. 2015;11(4):1–10. doi: 10.4018/ijec.2015100101

[54] Fornell C, Larcker DF. Evaluating structural equation models with unobservable variables and measurement error. J Mark Res. 1981;18(1):39–50. doi: 10.2307/3151312

[55] Jia Y, Cui L, Su J, Wu L, Akter S, Kumar A. Digital servitization in digital enterprise: leveraging digital platform capabilities to unlock data value. Int J Prod Econ. 2024;278:109434. doi: 10.1016/j.ijpe.2024.109434

[56] Hossain MA, Akter S, Yanamandram V, Strong C. Navigating the platform economy: crafting a customer analytics capability instrument. J Bus Res. 2024;170:114260. doi: 10.1016/j.jbusres.2023.114260

