# Supplementary material for: Dynamic service analytics capabilities and service firm performance: Exploring the mediating roles of adaptive and customer-linking capabilities
Source: PLoS One. 2026 Feb 6;21(2):e0338542. doi: 10.1371/journal.pone.0338542 (PMC12880647; doi:10.1371/journal.pone.0338542)
Supplement: S1 File — (DOCX) [file pone.0338542.s001.docx]

**S1 Table. Questionnaire items**

| Constructs | Measurement scales/items | Source |
| --- | --- | --- |
| Completeness(COM) | the dynamic service analytics used:  provide a complete set of information | [Akter et al.(2018)](file:///D:\\黄燕婷\\PhD\\博士期间\\paper\\动态服务分析能力\\1.服务分析能力对企业绩效的影响研究\\PloS%20one投稿\\动态服务分析能力如何影响服务公司绩效？——来自中国的经验证据-17.docx#_ENREF_5)，[Nelson et al.(2005)](file:///D:\\黄燕婷\\PhD\\博士期间\\paper\\动态服务分析能力\\1.服务分析能力对企业绩效的影响研究\\PloS%20one投稿\\动态服务分析能力如何影响服务公司绩效？——来自中国的经验证据-17.docx#_ENREF_36) |
|  | produce comprehensive information |  |
|  | all the information needed |  |
| Currency(CUR) | the dynamic service analytics used:  provide the most recent information |  |
|  | produce the most current information |  |
|  | always provide up-to-date information |  |
| Format([Fornell](#_ENREF_15)) | the information provided by the service analytics is well formatted |  |
|  | the information provided by the service analytics is well laid out |  |
|  | he information provided by the service analytics is clearly presented on the scree |  |
| Accuracy(ACC) | the dynamic service analytics used:  produce correct information |  |
|  | provide few errors in the information |  |
|  | provide accurate information |  |
| Connectivity(CON) | compared to rivals within our industry, our organization has the foremost available big data-driven service analytics system | [Akter et al.(2018)](file:///D:\\黄燕婷\\PhD\\博士期间\\paper\\动态服务分析能力\\1.服务分析能力对企业绩效的影响研究\\PloS%20one投稿\\动态服务分析能力如何影响服务公司绩效？——来自中国的经验证据-17.docx#_ENREF_5),  Kim et al.(2012),  Parasuraman et al.  (2005) |
|  | all remote, branch and mobile offices are connected to the central office for service analytics |  |
|  | our organization utilizes open systems network mechanisms to boost service analytics connectivity |  |
| Compatibility（COMP） | Software applications can be easily transported and used across multiple analytics platforms |  |
|  | our user interfaces provide transparent access to all platforms and applications |  |
|  | BDA-driven service insights is shared seamlessly across our organization, regardless of the location |  |
| Modularity(MOD) | reusable software modules are widely used in new analytics model development |  |
|  | end-users utilize object-oriented tools to create their own analytics application |  |
|  | object-oriented technologies are utilized to minimize the development time for new analytics applications |  |
| Privacy(PRI) | our big data-driven service analytics platform protects information about personal issues |  |
|  | our big data-driven service analytics platform protects information about personal identity |  |
|  | our big data-driven service analytics platform offers a meaningful guarantee that it will not share private information |  |
| Technical knowledge（TK） | our service analytics personnel are very capable in terms of programming skills | [Akter et al.(2018)](file:///D:\黄燕婷\PhD\博士期间\paper\动态服务分析能力\1.服务分析能力对企业绩效的影响研究\PloS%20one投稿\动态服务分析能力如何影响服务公司绩效？——来自中国的经验证据-17.docx#_ENREF_5),  Kim et al.(2012),  Tippins and Sohi.  (2003) |
|  | our service analytics personnel are very capable in terms of managing project life cycles |  |
|  | our service analytics personnel are very capable in the areas of data and network management and maintenance |  |
|  | our service analytics personnel create very capable decision support systems |  |
| Technology management knowledge(TM) | our service analytics personnel create very capable decision support systems |  |
|  | our service analytics personnel show superior understanding of technological trends |  |
|  | our service analytics personnel are very knowledgeable about the critical factors for the success of our organization |  |
|  | our service analytics personnel are very knowledgeable about the role of business analytics as a means, not an end |  |
| Business knowledge(BK) | our service analytics personnel understand our organization’s policies and plans at a very high level |  |
|  | our service analytics personnel are very capable in interpreting business problems and developing appropriate technical solutions |  |
|  | our service analytics personnel are very knowledgeable about business functions |  |
|  | our service analytics personnel are very knowledgeable about the business environment |  |
| Relational knowledge(RK) | our service analytics personnel are very capable in terms of planning, organizing and leading projects |  |
|  | our service analytics personnel are very capable in terms of planning, organizing and leading projects |  |
|  | our service analytics personnel are very capable in terms of teaching others |  |
|  | our service analytics personnel work closely with customers and maintain productive user/client relationships |  |
| Adaptive Capability(AC) | Meet a customer's demand changes in terms of service specifications | Lu et al.(2010),  Akter et al.(2021) |
|  | Tailor services according to a customer's request |  |
|  | Respond quickly to the demand for a service price change from a customer |  |
|  | The success rate of service improvement is high |  |
|  | The rate of service improvement is very fast |  |
|  | Satisfy the customer with a wide variety of strategies |  |
| Customer Linking（CL） | Establishing connections with new customers. | Motamarri et al.  (2022) |
|  | Enhancing communication with key target customers. |  |
|  | Maintaining connections with all the customers. |  |
| Firm performance(FP) | Using dynamic service analytics, we have improved customer retention compared to our competitors over the past three years | Tippins and Sohi  (2003) |
|  | Using dynamic service analytics, we have increased our sales performance compared to our competitors over the past 3 years |  |
|  | Using dynamic service analytics, we have increased our profitability compared to our competitors over the past 3 years |  |
|  | Using dynamic service analytics, our Return on investment has increased over the last 3 years compared to our competitors |  |
